# Does immunotherapy hold great promise in endometrial cancer care?

**DOI:** 10.3389/fimmu.2026.1763091

**Published:** 2026-03-16

**Authors:** Malgorzata Braszka, Hanna Chowaniec, Martyna Borowczyk, Ewa Dwojak, Maria Stępień, Antonina Ślubowska, Magda Mielczarek, Hanna Markiewicz, Rafał Ałtyn, Paweł Zieliński, Joanna Czerniak, Oliwier Adamczak, Andrzej Kluk, Grzegorz Dworacki, Paula Dobosz

**Affiliations:** 1 Cairns Base Hospital, Cairns, QLD, Australia; 2 Chair of Pathomorphology and Clinical Immunology, Poznan University of Medical Sciences, Poznan, Poland; 3 Department of Endocrinology, Internal Medicine and Metabolism, Poznan University of Medical Sciences, Poznan, Poland; 4 Department of Pathomorphology, University Clinical Hospital, Poznan, Poland; 5 Université Paris-Saclay, UVSQ, INSERM END-ICAP, Versailles, France; 6 Doctoral School, Medical University of Lublin, Lublin, Poland; 7 Cardinal Stefan Wyszynski University of Warsaw, Faculty of Medicine, Collegium Medicum, Department of Biostatistics and Research Methodology, Warsaw, Poland; 8 Department of Genetics, Wrocław University of Environmental and Life Sciences., Wrocław, Poland; 9 Department of Histology and Embryology, Faculty of Medicine, Medical University of Warsaw, Warsaw, Poland; 10 Department of Methodology, Faculty of Medicine, Medical University of Warsaw, Warsaw, Poland; 11 IT Department, Poznan University of Medical Sciences, Poznan, Poland; 12 Poznan University of Medical Sciences, Poznan, Poland

**Keywords:** adaptive cellular therapy, endometrial cancer, immunotherapy, o classification, oncovirus

## Abstract

Endometrial cancer (EC) is a hormonally driven malignancy with a strikingly uneven global distribution, interestingly occurring far more frequently in developed countries. Central to its pathogenesis is endocrine imbalance, which is most notably due to prolonged exposure to unopposed oestrogen, which fuels tumour initiation and progression. The dynamic interplay between oestrogen and progesterone signalling shapes disease biology and underpins the widespread use of hormonal therapies, particularly in early-stage disease and in patients who are not surgical candidates. Current EC management relies on a multimodal approach, integrating surgery, radiotherapy, hormonal therapy, and chemotherapy. However, the therapeutic landscape is rapidly evolving. Ongoing clinical trials are investigating innovative immunotherapeutic strategies, including biomarker-driven treatments, rational combination regimens, and adoptive cellular therapies. Immune checkpoint inhibitors have already demonstrated clinical benefit in mismatch repair–deficient EC. In parallel, cancer vaccines targeting tumour-associated antigens such as folate-binding protein (FBP), along with emerging modalities like CAR T-cell therapy, are being explored for their potential to reduce recurrence and improve long-term outcomes. Recent advances have highlighted the PI3K/AKT/mTOR signalling cascade as a key therapeutic target, offering opportunities to enhance the effectiveness of endocrine treatments. At the same time, growing evidence underscores the importance of crosstalk between hormonal dysregulation and immune mechanisms within the tumour microenvironment, a relationship that profoundly influences tumour behaviour and therapeutic response. In this review, we present a comprehensive overview of the current state of EC management and emerging therapeutic directions, with particular emphasis on treatment options available in Poland, the authors’ country of origin.

## 1 Introduction - demographics of endometrial cancer

The endometrial cancer incidence in the world varies significantly, both on the intra- and intercontinental levels. Most endometrial cancer cases are recorded in highly developed countries, with the so-called western lifestyle, where it is ranked the fourth most common cancer type in women (following breast cancer, lung cancer, and skin cancer) and the most frequent cancer of the reproductive organs (1). Disease incidence rates are the highest in North American countries (2). Undoubtedly, endometrial cancer incidence has been rising together with the average life expectancy length and obesity in these populations (1). The peak incidence is between 55 and 59 years of age, right after menopause (1). Endometrial carcinoma is rare in women under 40 years of age: the incidence in this age group has been variously reported to be from 1% to 8% of all cases of endometrial carcinoma (3). Risk factors, other than age, include: obesity, high blood pressure, diabetes, infertility or single childbirth, hormonal disorders caused by active ovarian tumours, long menstruation period, treatment by tamoxifen, and other comorbidities, such as Lynch syndrome (in the latter case, the risk is 40–60% higher throughout the lifespan) (4, 5).

Endometrial cancer quite early displays characteristic symptoms such as spotting and bleeding from birth canals. As the majority of cases occur after menopause, that symptom usually raises concerns in patients and makes them seek medical attention. Incidence of endometrial cancer is rising globally with the developed country seeing a rate of increase as high as 20% over 20 years, like in the USA (6). In Poland, an increased incidence has been observed since 1990, and this upward trend is expected to continue, along with a stable or slight increase in the mortality level (7).

## 2 Hormonal background and influence on cancer progression and treatment

The incidence of endometrial cancer is closely linked to hormonal imbalances (8). The interplay of oestrogen and progesterone, as well as other hormonal pathways, plays a pivotal role in both the development and progression of the disease (9).

Oestrogen promotes the proliferation of endometrial tissue (10), while progesterone acts as a counter-regulatory hormone, inducing cellular differentiation and inhibiting proliferation (11). Dysregulation in this balance, often due to excess oestrogen or insufficient progesterone, creates a permissive environment for the development of hyperplasia, a precursor to cancer (12). Primary sources of oestrogen include endogenous production in the ovaries (premenopausal) or peripheral conversion of androgens by aromatase in adipose tissue (postmenopausal) (13). Obesity, which increases aromatase activity, is a significant risk factor for endometrial cancer due to higher oestrogen levels (8). Additionally, conditions such as polycystic ovary syndrome (PCOS) and anovulation exacerbate oestrogen dominance by reducing progesterone exposure (14).

Hormonal imbalances also contribute to the molecular and cellular mechanisms that drive cancer progression (15). Elevated oestrogen levels activate oestrogen receptor alpha (ERα), which enhances cell proliferation, angiogenesis, and evasion of apoptosis in cancer cells (16). Conversely, the loss of progesterone receptor (PR) expression, common in advanced-stage tumours, diminishes the protective effects of progesterone and is associated with poorer outcomes (17). The strong association between unopposed oestrogen stimulation and endometrial cancer has led to the development of hormone-based therapies in endometrial cancer, particularly for early-stage disease or patients unsuitable for surgery (18). The most commonly used hormonal agents are progestins, which aim to counteract unopposed oestrogen by activating PRs to inhibit tumour proliferation and induce apoptosis (19). Progestins, such as medroxyprogesterone acetate (MPA) and megestrol acetate (MA), have shown efficacy in reducing tumour burden in low-grade, hormone receptor-positive cancers and are useful, especially in young women who want to preserve fertility (19). For advanced or recurrent disease, aromatase inhibitors (e.g., letrozole, anastrozole) (20)) and selective oestrogen receptor modulators (SERMs), such as tamoxifen (21), are considered, particularly in postmenopausal women. Emerging therapies targeting the PI3K/AKT/mTOR pathway, which is often activated in endometrial cancer, offer potential to enhance the efficacy of hormonal treatment (22).

Hormonal imbalance, particularly the predominance of unopposed oestrogen and the loss of progesterone signaling, not only drives the progression of endometrial cancer but also influences the tumour microenvironment (23, 24). It influences both innate and adaptive immune responses (25). This interplay between hormonal pathways and immune responses has critical implications for immunotherapy, which has emerged as a promising treatment modality in advanced or recurrent endometrial cancer (26). Oestrogen is known to modulate immune activity by promoting a tumour-supportive microenvironment (25). High levels of oestrogen upregulate the expression of programmed death-ligand 1 (PD-L1) on tumour and immune cells, facilitating immune evasion by inhibiting T-cell activity (27). Additionally, oestrogen influences myeloid-derived suppressor cells (MDSCs) and regulatory T cells (Tregs), enhancing their immunosuppressive roles within the tumour microenvironment (28).

Progesterone generally has anti-inflammatory effects and can inhibit immune responses by downregulating T-cell proliferation and cytokine production (28). Loss of progesterone receptor expression in endometrial cancer correlates with increased tumour aggressiveness and reduced immune surveillance (29). This oestrogen-dominant environment exacerbates immunosuppression, reducing the efficacy of immunotherapeutic strategies.

Beyond oestrogen and progesterone, metabolic hormones such as insulin and insulin-like growth factors (IGFs) also play a critical role in endometrial cancer progression. Hyperinsulinemia, frequently seen in obese patients, enhances chronic inflammation and creates an immune-suppressive state, promoting mitogenesis and anti-apoptotic signaling pathways, creating a microenvironment favorable for tumour growth (30). It can also diminish the efficacy of immune-based therapies.

## 3 Genetic and biological background

To understand the choices behind the clinical management of endometrial cancer, its genetic and biological background should be studied.

Contemporary classification and risk stratification are primarily based on molecular profiling introduced by The Cancer Genome Atlas (TCGA). Thanks to advances in research technology, including next-generation sequencing, assessment of microsatellite instability, and methylation profiling, TCGA presented a comprehensive molecular profile of EC based on 373 tumors, including endometrioid, serous, and mixed carcinomas. Four categories were established: 1) POLE ultramutated subgroup; 2) hypermutated group, microsatellite instability (MSI); 3) copy number-low, microsatellite stable (MSS) subgroup; 4) copy number high, serous-like tumours (31).

Somatic copy number alterations (SCNA) were shown to correlate with prognosis, with most serous and serous-like tumors demonstrating a high number of such alterations (31). Endometrioid carcinomas were characterized by frequent MSI, POLE mutations, and activation of WNT/CTNNB1 signaling. Serous carcinomas commonly exhibited non-silent TP53 mutations, high-volume SCNA, ERBB2 amplification (27%), and PIK3CA mutations (42%) (31).

The validated ProMisE (Proactive Molecular Risk Classifier for Endometrial Cancer) system, a continuation of the TCGA project, further developed molecular subtyping by separating EC into four prognostically distinct subtypes: POLE-mutated (POLEmut), mismatch repair deficiency (dMMR), p53 wild-type (p53wt), and p53 abnormal (p53abn) (32). More recent classification divides EC into: POLE ultramutated, microsatellite instability (MSI) hypermutated, copy number low, copy number high. Additionally, the presence of HER2 and LVSI have also been shown to carry prognostic value in EC (33).

The division of EC was proposed by Bokhman in 1983 who split its pathogenicity into two types (34)Type I (around 70% of cases) is described as one presenting in correlation with obesity and metabolic syndromes like hyperlipidaemia and diabetes. It stems from endometrial hyperplasia driven by factors like hypoestrogenism and is associated with a better prognosis. Type II (around 30% of EC cases) has been described as arising from atrophic endometrium and a worse prognosis defined by poorly differentiated tumours and increased metastasis rates (35).

Further, histopathological characteristics divide EC into the following types: serous carcinoma and clear cell carcinoma (mostly seen in type II), carcinosarcoma, endometrioid adenocarcinoma (mostly seen in type I). Wilczynski et al., 2016 noted the oversimplification of the Bokhman’s division which does not correspond to the clinical evidence of EC pathology (35). It has been suggested that mutations involved in the neoplasticity of type I EC involve PI(3)K/AKT (phosphatidylinositol-3-OH) pathways (36, 37); *FGFR2*, *ARID1A*, *CTNNB1*, *PIK3CA*, *PIK3R1* and *KRAS* with microsatellite instability (MSI) responsible for 30% of them (38, 39).

## 4 State of the art - how do we treat endometrial cancer today

The main treatments for endometrial cancer include surgery, radiotherapy, hormone therapy, and chemotherapy. In Poland, the primary treatment is often a total hysterectomy with bilateral salpingo-oophorectomy, sometimes along with pelvic and para-aortic lymphadenectomy (39, 40). Radiation therapy is used post-operatively in cases with a high risk of recurrence, particularly in high-grade tumours or with deep myometrial invasion (41). For certain cases, especially in younger women or those with specific tumour types (e.g. ovarian endometrioid carcinoma) hormone therapy is used. Chemotherapy is usually considered for advanced or recurrent cases. Common regimens include carboplatin and paclitaxel.

Treatment approaches are similar across the European Union (EU), with an emphasis on guidelines by the European Society of Medical Oncology (ESMO). Newer therapies that target specific pathways in endometrial cancer are becoming more common, such as immunotherapy with pembrolizumab and durvalumab approved for mismatch repair-deficient endometrial cancers (42). Each country may have specific treatment guidelines, influenced by local expertise, but generally aligned with ESMO recommendations (43). In global medicine, surgery remains the cornerstone of treatment around the world, although approaches to radiotherapy and chemotherapy may vary.

**Figure 1:**
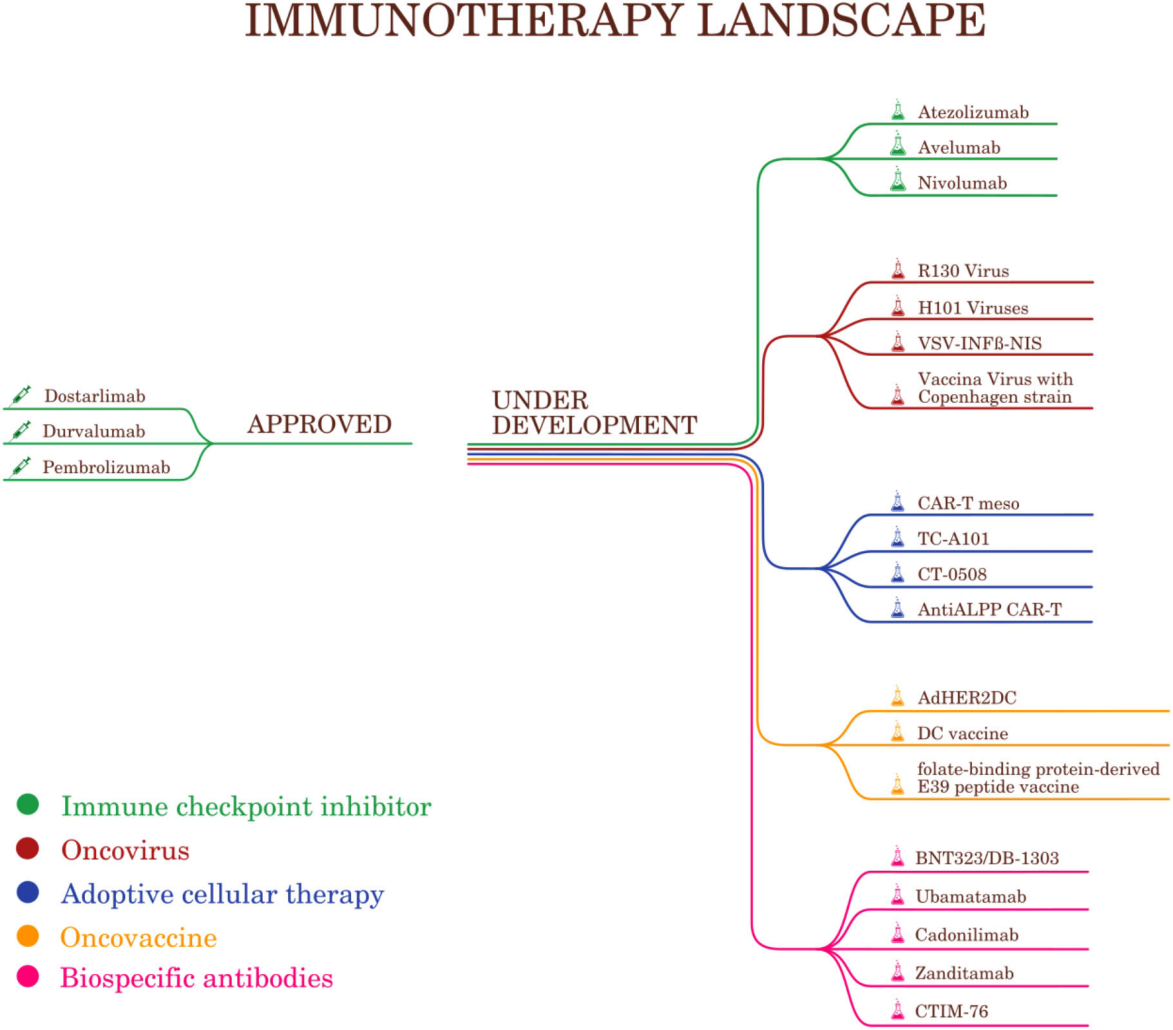
Landscape of immunotherapy in endometrial cancer treatment.

The most recent U.S. Food and Drug Administration (FDA) and European Medicines Agency (EMA) approvals in immunotherapy for EC treatment, include pembrolizumab and dostarlimab, followed by durvalumab for mismatch repair-deficient cancers, reflecting a significant shift toward personalized medicine (**Figure 1**) (44, 45). Standard chemotherapy combinations, such as carboplatin and paclitaxel, remain widely accepted and are considered the backbone of treatment for endometrial cancer. However, to improve treatment outcomes, many investigational drugs and drug combinations are currently undergoing clinical trials. These may include new immunotherapies or therapies targeting specific genetic markers. These include not only next-generation immunotherapies but also therapies targeting specific genetic and molecular alterations identified through genomic profiling. Such agents are directed against HER2 overexpression for example trastuzumab in HER2-positive serous endometrial cancer (46) (47), PI3K/AKT/mTOR pathway inhibitors for tumors harboring PIK3CA or PTEN alterations (48) (22) and PARP inhibitors in tumors with homologous recombination deficiency (49). Additionally, antibody–drug conjugates and combinations of immune checkpoint inhibitors with targeted therapies are being evaluated to enhance antitumor activity and overcome resistance mechanisms (47–49).While not yet FDA or EMA approved, these investigational agents show promise in enhancing therapeutic efficacy and addressing unmet clinical needs. The treatment landscape for endometrial cancer is becoming increasingly individualized, driven by advances in genetic profiling and the identification of molecular subtypes. Ongoing research continues to expand treatment options and refine therapeutic strategies to optimize outcomes for patients. It is important to note that treatment approaches can vary significantly based on geographical region, availability of resources, and access to clinical trials. These factors play a critical role in determining the feasibility and implementation of advanced therapies in different healthcare settings.

## 5 Potential of immunotherapy and types of immunotherapy

### 5.1 Immune checkpoint inhibitors

Programmed cell death protein 1 (PD-1) is a protein receptor expressed on the surface of T lymphocytes. When PD-1 binds to its ligand programmed cell death protein 1 ligand (PD-L), which is overexpressed on tumour cells, the immune response is suppressed, and apoptosis of tumour cells is inhibited. Antibodies targeting this pathway prevent the interaction between PD-1 and PD-L1, thanks to this the immune system can act against tumours (45). PD-1 inhibitors include nivolumab, pembrolizumab, and dostarlimab, while PD-L1 inhibitors include atezolizumab, avelumab, and durvalumab. It is important that PD-L1 inhibitors, which target ligands found on tumour cells, may be associated with fewer immune-related adverse effects compared to PD-1 inhibitors. PD-L1 inhibitors do not block the interaction between PD-1 and PD-L2. PD-L2 is expressed on the surface of hematopoietic cells and plays a distinct role in regulating immune responses. In contrast, PD-1 inhibitors completely block the binding of both PD-L1 and PD-L2 to PD-1, potentially leading to broader immune modulation (50). **Table 1**. presents a summary of FDA-approved checkpoints inhibitors.

**Table 1:** Approved immune checkpoint inhibitors and their indications by FDA and EMA.

Treatment regimen	Brand name	Type of treatment	Indication FDA	Indication EMA
Pembrolizumab	Keytruda	Monotherapy	Advanced, unresectable, or metastatic endometrial cancer dMMR/MSI-H[+] progression after previous systemic treatments (51)	Advanced or recurrent endometrial carcinoma dMMR/MSI-H[+] progression on or following prior treatment with a platinum containing therapy, that are not candidates for curative surgery nor radiation (52)
	Keytruda	With Lenvatinib	Advanced endometrial cancer dMMR/MSI-H[-] (53)	Advanced or recurrent endometrial carcinoma progression on or following prior treatment with a platinum containing therapy, that are not candidates for curative surgery nor radiation (52)
	Keytruda	With chemotherapy	Advanced or recurrent endometrial cancer (54)	First-line treatment in primary advanced or recurrent endometrial carcinoma (52)
Durvalumab	Imfinzi	With chemotherapy	Advanced or recurrent endometrial cancer dMMR[+] (55)	Advanced or recurrent endometrial cancer for initial treatment; For maintenance treatment in monotherapy dMMR[-] and in combination with olaparib for dMMR[+] (56)
Dostarlimab-gxly	Jemperli	With chemotherapy followed by single-agent dostarlimab	Primary advanced or recurrent endometrial cancer with or without dMMR/MSI-H (57)	Advanced or recurrent endometrial cancer suitable for systemic therapy (58)

#### 5.1.1 Pembrolizumab

Pembrolizumab blocks the PD-1/PD-L1 pathway. In 2021 it was approved in monotherapy in patients with advanced, or metastatic endometrial cancer characterized by high microsatellite instability (MSI-H) or mismatch repair deficiency (dMMR), when the disease has progressed after previous systemic treatments in patients not suitable for surgery or radiation. This approval was based on efficacy results from the KEYNOTE-158 study (51).

In 2024, pembrolizumab was approved by the FDA in combination with and without chemotherapy for adult patients with primary advanced or recurrent endometrial cancer (54). Its efficacy was evaluated in KEYNOTE-868/NRG-GY018 (59), which showed that progression-free survival in the dMMR cohort was 74% in the treatment group and 38% in the placebo group (60).

Several other ongoing clinical trials are investigating pembrolizumab for the treatment of endometrial cancer, often in combination with chemotherapy or other treatments. A notable example of these trials is KEYNOTE-B21/ENGOT-en11/GOG-3053 Trial, which examines pembrolizumab with adjuvant chemotherapy, with or without radiotherapy, in patients with newly diagnosed endometrial cancer who have undergone surgery with curative intent (61). Although it was not proven that pembrolizumab improves disease-free survival in newly diagnosed patients with high-risk, all-comer endometrial cancer, the results suggested that pembrolizumab combined with chemotherapy improved disease-free survival in patients with dMMR tumours (62).

The efficacy of the lenvatinib plus pembrolizumab combination was provided by the phase Ib/II KEYNOTE-146 (Study 111) trial, which evaluated this regimen in patients with previously treated advanced endometrial cancer. The study demonstrated durable antitumor activity in the pMMR population. In the overall cohort, the objective response rate was 39.8%, with a median duration of response of 22.9 months. Median progression-free survival and overall survival were 7.4 months and 17.7 months, respectively. Long-term follow-up confirmed sustained efficacy with a manageable safety profile, supporting further evaluation of this combination in the phase III KEYNOTE-775 trial (63).

The approval of this regimen by the FDA was based on the results of the phase III KEYNOTE-775 (Study 309) trial, which compared lenvatinib plus pembrolizumab with chemotherapy. The study demonstrated a significant improvement in both progression-free survival and overall survival with the combination therapy. In the pMMR population, median progression-free survival was 6.6 months in the lenvatinib plus pembrolizumab group compared with 3.8 months in the chemotherapy group, while median overall survival was 17.4 months versus 12.0 months, respectively (64).

#### 5.1.2 Durvalumab

Durvaluamb is a monoclonal antibody that inhibits the interaction of PD-1/PD-L1. It is FDA-approved in combination with chemotherapy to treat advanced endometrial cancer with dMMR (65). Its efficacy was evaluated in DUO-E study which recruited primary advanced or recurrent endometrial cancer patients. A significantly lower risk of disease progression or death when durvalumab was integrated into the treatment regimen was demonstrated (66).

#### 5.1.3 Dostarlimab

Dostarlimab is an IgG4-k antibody that targets PD-1, preventing its interaction with PD-L1 and PD-L2 (67). Dorsalimab was initially approved by the FDA for patients with dMMR or MSI-H endometrial cancer. In 2024, its indication was expanded as treatment in combination with chemotherapy and followed by dostarlimab maintenance monotherapy for patients with primary advanced or recurrent endometrial cancer regardless of their dMMR status. Its expanded indication stems from the completion of the Phase 3 RUBY trial which proved the efficacy of the regimen of dostarlimab and chemotherapy in lowering the risk of progression or death compared to placebo with 72% lower risk in the dMMR-MSI-H population and 36% lower risk in the overall population (67).

Ongoing trials such as Phase 3 DOMENICA (68), are investigating the efficacy of dostarlimab in first-line advanced and metastatic dMMR deficient endometrial cancer compared to chemotherapy. The SATELLITE study evaluates dostarlimab potential as a non-surgical option for those unwilling or unsuitable to undergo surgical interventions in early-stage endometrial cancer with dMMR. Its primary completion is estimated in 2026 (69).

#### 5.1.4 Nivolumab

Nivolumab is a monoclonal antibody that selectively binds to the PD-1 receptor. Phase 2 study investigated its efficacy in dMMR and MSI-H positive and hypermutated tumours patients. Nivolumab monotherapy showed clinical activity in these subtypes, however around 60% of the studied population failed to respond to it or had progression of disease in 6 months (70). Another phase 2 study showed that a combination of nivolumab and cabozantinib in the treatment of recurrent, advanced and metastatic EC resulted in improved outcomes in those who received previous immunotherapy. In the nivolumab and cabozantinib group the median PFS was 5.3 (90% CI 3.5 to 9.2) months (n=36) and 1.9 (90% CI 1.6 to 3.4) months in those treated only with nivolumab alone (n=18) (HR = 0.59, 90% CI 0.35 to 0.98) (71). Another Phase II trial NRG-GY025 is comparing nivolumab in monotherapy and combination with ipilimumab for dMMR recurrent endometrial cancer, aiming to assess whether combined checkpoint inhibition offers superior results (72).

In 2023 a phase II clinical trial NCT05795244 was initiated and it is investigating the effectiveness of nivolumab in patients with surgically resectable dMMR endometrial cancer. It aims to evaluate the impact of nivolumab on post-surgical outcomes in endometrial cancer patients dMMR (73).

#### 5.1.5 Avelumab

Avelumab is a monoclonal antibody that binds to PD-L1. When studied in combination with chemotherapy, it resulted in improvement in progression-free survival (PFS) in patients with advanced and recurrent endometrial cancer in phase 2–2 MITO END-3 trial (74). A combination of avelumab and talazoparib, which is a poly-ADP ribose polymerase inhibitor used in the treatment of breast cancer, has been evaluated in a Phase 2 study which indicated a favourable profile of the regimen in patients with recurrent MMRP EC (75). A new phase 2 study was initiated in 2024 to investigate the effect of avelumab in combination with ATR inhibitor (M1774) in patients with recurrent endometrial cancer previously treated with immunotherapy (76).

#### 5.1.6 Atezolizumab

Atezolizumab, as a monoclonal antibody targets PD-L1. The AtTEnd study (77) recruited 549 patients with advanced or recurrent endometrial cancer, or notably carcinosarcoma who were assigned to receive either atezolizumab or a placebo plus chemotherapy (78). The endpoints included PFS and overall survival (OS), both in the overall population and in the dMMR subgroup. In the dMMR group, the median PFS was not reached in the atezolizumab group (95% CI: 12.4–NE), but it was 6.9 months in the placebo group (HR: 0.36; p = 0.0005). In the overall population, the median PFS was 10.1 months in the atezolizumab group compared to 8.9 months in the placebo group (HR: 0.74; p = 0.022). Adding atezolizumab to chemotherapy improved PFS, particularly in patients with dMMR tumours (including carcinosarcoma). That suggests, this combination may be beneficial as a first-line treatment for this specific subgroup of patients (78).

### 5.2 Oncological vaccines

With the growing popularity of ani-HPV vaccination, there is a natural shift in scientific interest in exploring new therapeutic possibilities in gynecological cancers. Cancer vaccines’ therapeutic profile enhances the body’s adaptive immune system response to malignant cells. Recurrence prevention as the main goal of vaccine studies has shown its promise while targeting folate-binding protein (FBP) commonly expressed on malignant cells as described in the phase I/IIA trial by (79). The E39 peptide vaccine demonstrated the ability to prevent recurrence in high-risk endometrial cancer patients. However, it is also proof that the biggest effectivity was achieved in patients previously receiving treatment for primary disease and with low FBP expression.

Oncological vaccines can be divided depending on their mechanism of action spanning from peptides and proteins, whole tumour cells, and nucleic acid bases to dendritic cells. The last one is the most researched form of vaccine in the treatment of endometrial cancer. Harari et al., 2021 (80) investigated the combination of dendritic cell vaccine pulsed with peptide neoantigens as an adjunct to standard care regime (systemic chemotherapy) in serous MMR, p-53 endometrial inoperable cancer recurrence. They demonstrated that a personalized vaccination can be created using autologous monocyte-derived dendritic cells and lead to potent, polyfunctional, and durable T-cell responses which correspond to clinical benefit in the disease. Furthermore, in a prospective single-arm phase I/II study (81),studied 7 patients with metastasis EC who all expressed both Survivin and Mucin-1 antigen on their tumour material and their response to DC vaccination combined with platinum-based chemotherapy. The administration of the DC vaccine involved ultrasound-guided intranodal injection to eliminate the risk of not reaching the lymph nodes involved in intravenous or intradermal injections. This study, although small, proved that administration of the combined treatment was possible and safe, however, the efficacy remained to be further proven. Currently, the Phase 1/2 study is investigating a dendritic vaccine (FRalphaDC) in combination with pembrolizumab in high-grade serious, endometrioid, and clear cell carcinoma with high expression of FRalpha (82). Its completion is estimated in 2027.

Additionally, there is a growing interest in AdHER2DC vaccine in HER2-expressing endometrial cancer. A study announced in 2024, investigates AdHER2DC together with ANKVITA (IL-15 superagonist immune enhancer, approved by FDA in treatment for bladder cancer in April 2024), pembrolizumab, and lenvatinib (83). The study will evaluate 60 subjects and is scheduled to be completed in 2026 (84).

Despite the volume of the ongoing research initiatives, limitations of oncovaccines like the variable ability to elicit a rapid and strong T-cell response, evaluation of the target antigen or even defining the target antigen, should be recognized.

### 5.3 Oncoviruses

There is a growing number of pre-clinical and early clinical studies focusing on the development of oncoviral therapies targeting endometrial cancer.

One of them is the study conducted by Liu et al., 2014 demonstrating that Type I and Type II endometrial cancer is susceptible to oncolysis upon exposure to vaccinia virus (VV) with Copenhagen strain being more effective in its oncolytic effect (85). When studied *in vitro*, cell lines of Type II EC were more effectively killed by VV than Type I EC. With Type II EC showing a higher mortality profile, further development of VV can serve as a therapeutic promise to those diagnosed, however, the Copenhagen strain’s side effect profile should be noted with some of the mice developing pox lesions upon exposure.

Phase 1 study investigated the administration of VSV-IFNβ-NIS monotherapy, and in combination with Avelumab patients with a refractory solid tumour, including endometrial cancer with significant evidence of anti-tumour activity (86, 87). Phase 2 of the study was announced in 2020 at the ASCO Annual Meeting, however up to date, there has been no release of the study results (88). Simultaneously, a Phase 1 trial of VSV-hIFNbeta-NIS with or without ruxolitinib phosphate in stage IV endometrial cancer or recurrent endometrial cancer has been initiated and its recruitment completed however results have not yet been published (89).

Recruitment for early Phase 1 study is currently ongoing for investigations of efficacy and safety of R130 virus (recombinant herpes simplex virus I) in patients with relapsed and refractory endometrial cancer. The oncolytic recombinant induces T-cell toxicity. The study is estimated to involve 20 participants and its results are expected in 2026 (90).

Similarly, the Phase 2 clinical trial is an investigation of intra-tumour injection of H101 oncolytic viruses combined with or without radiotherapy in refractory or recurrent endometrial cancer. The study is active, but not yet recruiting (88, 89).

The growing body of research on oncoviral therapy in endometrial cancer is remarkable, however further evidence is needed regarding its clinical potential and safety.

### 5.4 Adoptive cellular therapy

Adoptive cellular therapy in endometrial cancer refers to the use of modified immune cells, such as chimeric antigen receptor (CAR) T-cells, to enhance the immune system’s ability to target and destroy cancer cells. The therapy involves extracting a patient’s T-cells, modifying them to recognize specific tumour antigens, and then reinfusing them back into the body. While early studies show potential, challenges remain, such as the need to overcome the immunosuppressive tumour microenvironment of endometrial cancer and improve treatment efficacy and safety for broader patient populations. Further clinical trials, discussed below, are ongoing to evaluate its full potential (91).

In 2021, Phase I, the first-in-human study of adenovirally transduced autologous macrophages engineered to contain an anti-HER2 chimeric antigen receptor was initiated by Reiss et al. (92) (93, 94). This trial was carried out to evaluate the safety, tolerability, and manufacturing potential of CT-0508, an autologous macrophage engineered to contain an anti-HER-2 CAR. Patients diagnosed with HER-2-overexpressing solid tumours who have failed conventional treatment were included (95). Furthermore, CAR-T cells that target anti-alkaline phosphatase placental (ALPP) were studied in the First-in-Human Anti-ALPP CAR-T Cells Immunotherapy for Ovarian and Endometrial Cancer clinical trial (96). The study aimed to evaluate the cases of ALPP-positive subjects who experienced treatment-related adverse effects after the infusion of TC-A101 as well as to assess the overall response rate (ORR) to TC-A101 infusion over eight weeks and the number and percentage of ALPP-CAR-T cells in the circulatory system from ALPP-positive patients after six months of treatment. Another trial in Phase I NCT02580747 focused on the treatment of relapsed and/or Chemotherapy Refractory Advanced Malignancies. The efficacy of chimeric mesothelin antigen receptor-modified T (CAR-T-meso) cells was investigated (97). It was assumed that using genetically engineered tumour-specific CARs into autologous or donor T boosts the immune system.

Nevertheless, very limited clinical trials are investigating CAR-T cell therapy for endometrial cancer in Poland. The Polish Chimeric Antigen Receptor T-cell Network, funded by the Medical Research Agency (MRA), is an initiative aimed at developing CAR-T therapies in Poland (98). This project focuses on enhancing the production and accessibility of CAR-T treatments across the country. Although the initial focus of this project is on hematological cancers, the infrastructure being developed could facilitate future trials for solid tumours, including endometrial cancer.

### 5.5 Bispecific antibodies

Bispecific antibodies (bsAbs) are genetically applied solutions that may result in antigenic consequences. Bispecific antibodies can be designed to recognize antigens specific to selected cancer (99); for example: EpCAM (adhesion antigen) – is present on the surface of many cancer cells, HER2 – in cancer subtypes associated with overexpression of this protein, PD-L1 – a protein that allows cancer to avoid attacks by the immune system.

**Figure 2:**
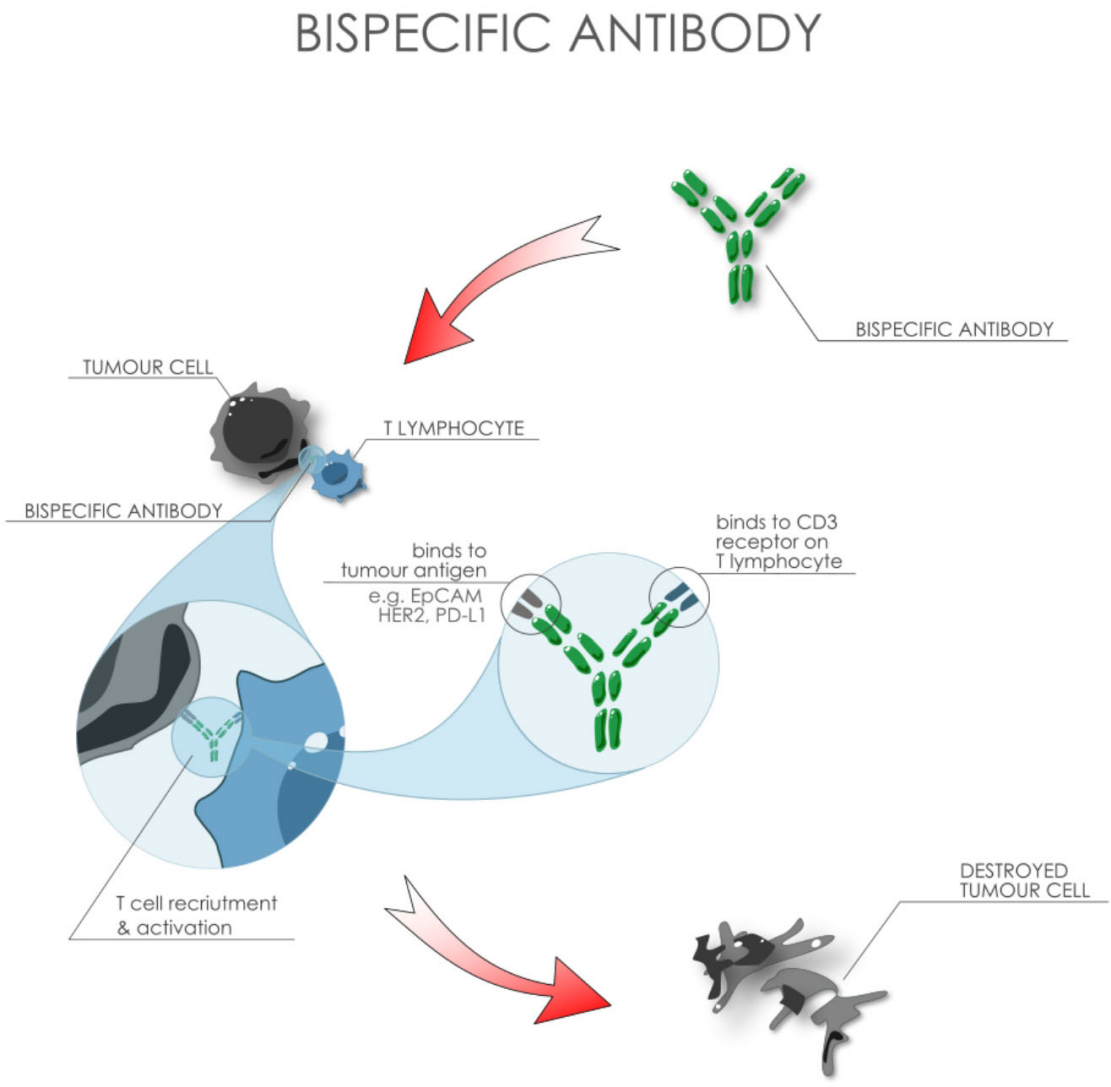
Mechanism of action of biospecific antibodies.

One fragment of the bispecific antibody binds to the antigen on the cancer cell, and the other engages the immune system, e.g. by binding to the CD3 receptor on T lymphocytes (**Figure 2**). T lymphocytes can be “led” by bsAbs directly to cancer cells, resulting in their elimination. Compared to traditional methods such as chemotherapy or radiotherapy, bispecific antibodies can target highly specific features of the tumour, minimizing damage to healthy tissue and reducing side effects (100). Currently, over 200 bsAbs, with increasingly diverse structures and mechanisms of action, are in the preclinical or clinical development phase for the treatment of various tumours (101).

Bispecific antibodies offer several advantages in EC treatment. They provide a treatment option for cancers resistant to other therapies, enhance the immune system’s ability to target cancer cells more effectively, and have fewer side effects compared to traditional treatments like chemotherapy. Additionally, they can be combined with hormone therapy to achieve a synergistic effect. However, their full potential is still to be further studied as per ongoing clinical trials described below.

#### 5.5.1 BNT323/DB-1303

BNT323/DB-1303 is a biospecific antibody that targets HER2. In 2023 it was granted a Breakthrough Therapy designation by FDA for the treatment of advanced endometrial cancer in those who progressed on or after treatment with immune checkpoint inhibitors (102). Such a decision came after the publishing of the Phase 1/2 results in which DB-1303 showed a high response rate and positive safety profile (103).

#### 5.5.2 Ubamatamab

Ubamatamab bridges MUC16 (cell surface glycoprotein) and T cells and is currently being investigated in a Phase 2 trial for patients with endometrial cancer who show overexpression of MUC16 (104).

#### 5.5.3 Cadonilimab

Cadonilimab was studied in combination with lenavatinib in patients with advanced endometrial cancer who previously received one or more platinum-based chemotherapies and showed favorable results for this population (105).

#### 5.5.4 Zanditamab

Zanditamab (ZW25) is currently being investigated in the Phase 2 study for patients with HER2-expressing tumours, including endometrial neoplasm (106), despite a previous Phase 2 study showing low response to the drug in recurrent HER2+ endometrial carcinoma and carcinosarcomas (107).

#### 5.5.5 CTIM-76

Phase 1 trial is actively ongoing for CTIM-76 (a CLDN6 x CD3 T cell engaging bispecific antibody) investigating its safety and efficacy in CLDN6-positive advanced or metastatic endometrial cancer (108).

## 6 Challenges for immunotherapy and understanding tumour microenvironment

Immunotherapy in endometrial cancer faces multiple challenges, including the complexity of the tumour microenvironment, which is influenced by hormonal fluctuations and immune tolerance mechanisms. Variability in tumour-infiltrating lymphocytes (TILs), their location, and their interaction with other immune cells further complicate the prediction of therapy success. Additionally, the presence of tumour-associated macrophages (TAMs) contributes to immune suppression and poor prognosis, complicating treatment.

The tumour microenvironment (TME) in endometrial cancer presents a complex challenge for immunotherapy, balancing immune defense and tolerance. The endometrial immune system must uniquely protect against sexually transmitted infections while simultaneously accommodating the growth of an allogeneic foetus during pregnancy. This dual role is tightly regulated by sex hormones, which modulate immune activity in the female reproductive tract. Immune cells, such as natural killer (NK) cells, macrophages, and adaptive lymphocytes, display cyclical changes in number and function throughout the menstrual cycle, reflecting their role in host defense and tissue remodeling. These hormonal shifts create a dynamic and sensitive environment, complicating the design of immunotherapeutic strategies. Furthermore, endometrial carcinogenesis is impacted by different stromal cell populations with their unique functions.

Furthermore, myofibroblasts secrete growth factors that contribute to EC progressions, together with increased angiogenesis, secretion of VEGF, and metastasis rates. Similarly, by secreting factors like hepatocyte growth factor (HGF), CXCL12 myofibroblasts promote EC growth and invasion (109). Macrophages are another group of immune cells that play a crucial role in the TME of EC. Among them, the different subtypes of macrophages have been noted to cause antimural effects (M1) and tumour progression promotion (M2) (110). EC’s environment shows higher levels of macrophages in their environment than non-cancerous endometrium (111). Macrophages are located in suboptimal oxygenation tissues and they produce pro-inflammatory cytokines and oxygen free radicals enabling further angiogenesis (109). Interestingly, the infiltration of the immune cells and the pattern of their distribution (112) has been closely monitored in EC. Similarly, NK cell inflatiraiton has shown an association with better cancer survival (113). Increased infiltration of intramular CD8+ T-cells has been previously associated with better prognosis with its level varying between different molecular subtypes of EC (114). Stromal signalling has been widely described in the pathogenesis of EC. Among them, the extracellular matrix that plays a role in EC pathogenesis and has a role in EC-promoting TGF-β signaling pathway has also been described (115). Protein mutations, including adenomatous polyposis coli (APC), have been shown to drive the increase in stromal myofibroblasts, oestrogen, progesterone receptors, and increased angiogenesis (116). Similarly, stromal signaling of pathways such as LKB1 (117), HDN2 (118), and VEGF (119)have been investigated. There is clear evidence in the literature highlighting the effect of unopposed oestoregen on the tumourigenesis of EC (115).

Obesity, being a known risk factor for the development of EC, is linked with an increased amount of adipose tissue which secretes high amounts of growth factors and adipokines which have the potential to increase tumour cell growth and invasion (120)by promoting secretion of adipokines, IGF-1, insulin, oestorgen (109). Adipocytes also secrete leptin, a protein that has been linked with pro-angiogenic factors (121). Furthermore, the altered hormonal balance in obesity increases pro-inflammatory cytokines levels and drives insulin resistance, further increasing the availability of IGF-1, which promotes EC proliferation (122). Furthermore, obesity promotes the endogenous synthesis of sex hormones, including oestorgen fuelling endometrial hyperplasia (115).

Understanding how this immune-hormonal interplay affects tumour progression and therapy response is crucial for advancing treatment in endometrial cancer (123).

### 6.1 Balancing hormones in immunotherapy

The interplay between hormonal imbalance and immune dysregulation presents challenges and opportunities for the application of immunotherapy in endometrial cancer (124).

Hormonal imbalance, particularly oestrogen-mediated PD-L1 expression, may influence the response to immune checkpoint inhibitors, such as pembrolizumab and dostarlimab, targeting the PD-1/PD-L1 axis (125). Identifying patients with high PD-L1 expression and hypermutated tumours, such as those with microsatellite instability-high (MSI-H) or mismatch repair-deficient (dMMR) profiles, is critical for selecting candidates likely to benefit from these agents (126), as such patients may respond better to pembrolizumab (127).

Combining hormonal therapies with immunotherapy may overcome the immunosuppressive effects of hormonal imbalance (128). Restoring progesterone signaling may enhance anti-tumour immunity by modulating the immune microenvironment and counteracting oestrogen-driven immunosuppression (129). By reducing systemic oestrogen levels, aromatase inhibitors could reduce PD-L1 expression and improve immune responses when used alongside immune checkpoint inhibitors such as in the case of breast cancer (130).

Therapies aimed at reducing chronic inflammation and metabolic dysfunction associated with obesity and hyperinsulinemia could synergize with immunotherapy, enhancing its effectiveness, such as an addition of metformin to immunotherapy (131). A phase I study of temsirolimus in combination with metformin in patients with advanced or recurrent endometrial cancer showed that metformin can be safely combined with temsirolimus, offering a modest therapeutic benefit without increasing safety risks (132).

Clinical trials investigating the combination of hormonal agents with immune checkpoint inhibitors or other immunomodulatory therapies hold promise for improving outcomes in patients with hormonally driven and immunologically active tumours.

### 6.2 Tumour infiltrating lymphocytes and their impact on the prognosis of survival

Research into tumour-infiltrating lymphocytes (TILs) in endometrial cancer has shown mixed results regarding their prognostic value, particularly the role of CD8+ T cells, which are important for cytotoxic immune responses. Some studies suggest that a higher density of CD8+ TILs is associated with better overall survival (OS) and disease-free survival (DFS) in endometrial cancer patients, especially in Type I tumours, which tend to have a more favorable immune microenvironment​. However, these findings are not consistently observed across all patient cohorts. In more aggressive cancers, particularly Type II endometrial cancers, the presence of CD8+ TILs does not always correlate with improved survival outcomes (133). Moreover, the impact of TILs varies depending on tumour grade and mismatch repair (MMR) status. For example, in high-grade endometrial cancer, TILs were linked to better progression-free survival, whereas no effect was seen in low-grade tumours (127). In some studies, intraepithelial TILs near invasive margins correlate with improved survival (134), while perivascular lymphocytic infiltrates have been associated with poorer outcomes (135). Given the complexity of the immune landscape in endometrial cancer, the predictive value of TILs requires more research to clarify their role in different tumour subtypes and to refine their use as biomarkers for survival.

### 6.3 T cell exhaustion and anergy targeted by modern immunotherapy strategies

T cell exhaustion is a dysfunctional state that arises when T lymphocytes, particularly CD8^+^ cytotoxic T cells, are chronically stimulated by persistent antigen exposure and immunosuppressive signals within the tumor microenvironment (TME). In this state, exhausted T cells show progressive loss of effector functions such as cytokine production and cytotoxicity, coupled with sustained upregulation of multiple inhibitory receptors (e.g., PD-1, LAG-3, TIM-3), altered transcriptional programs, and impaired proliferative capacity, which collectively limit their ability to control tumor growth and contribute to resistance to immunotherapies such as immune checkpoint inhibitors (ICIs) and adoptive cell therapies (136). In cancers broadly, and by extension in endometrial carcinoma where immune surveillance and neoantigen load vary across molecular subtypes, exhausted T cells correlate with poor responses to ICIs like anti-PD-1 therapies despite their clinical benefit in mismatch repair-deficient or POLE-mutant tumors, suggesting that exhaustion-linked mechanisms are relevant to both response and resistance in this disease (135, 136).

Overcoming T cell exhaustion is a major focus of next-generation immunotherapy strategies. Clinically established approaches such as PD-1/PD-L1 blockade aim to reinvigorate exhausted T cells by disrupting inhibitory receptor signaling, thereby restoring some effector function and proliferative potential within a subset of progenitor-like exhausted cells (137). Beyond checkpoint inhibition, emerging strategies under investigation include epigenetic reprogramming (e.g., DNA methyltransferase or histone deacetylase inhibitors) to reset exhausted T-cell transcriptional states and enhance responsiveness to immunotherapy, metabolic interventions to improve T-cell fitness in nutrient-poor TMEs, and engineered cell therapies (such as optimized CAR-T or TCR-T cells) designed to resist exhaustion programs (138, 139). Rational combination regimens that pair ICIs with epigenetic, metabolic, or adoptive cell therapy modalities are also being explored to broaden efficacy and overcome multiple layers of exhaustion-mediated resistance, with potential applicability to immunotherapy-resistant subsets of endometrial cancer (137).

Moreover, T cell anergy is a hyporesponsive state in which T cells remain alive but fail to proliferate or exert effector functions after antigen engagement, typically because they receive insufficient co-stimulatory signals during T cell receptor (TCR) activation, leading to impaired IL-2 production and downstream signaling dysfunction (137, 140). In the context of cancer, anergy can be induced by the immunosuppressive tumor microenvironment (TME), characterized by high expression of inhibitory ligands and low costimulation, which together promote T cell unresponsiveness and limit effective anti-tumor immunity (141–143). This anergic state contributes to resistance to immunotherapies, especially those that depend on reactivating effector T cells such as checkpoint inhibitors or adoptive T cell therapies, because these cells lack the baseline responsiveness necessary for reactivation (143). In endometrial cancer, where T cell activation status is a key determinant of treatment response, anergy may further diminish the pool of functional T cells, compounding other dysfunctions like exhaustion and reducing clinical efficacy of immunotherapeutic approaches (144). Strategies under investigation to overcome anergy include enhancing co-stimulation (e.g., via CD28 agonists), modulating NFAT-dependent transcriptional programs, and combining therapies that improve antigen presentation and warm up T cell activation thresholds, thereby restoring responsiveness and potentially improving immunotherapy outcomes (143, 145).

### 6.4 Tumour-associated macrophages impact on recurrence-free survival

Tumor-associated macrophages (TAMs) are increasingly recognized as key players in shaping the tumor microenvironment (TME) of endometrial cancer, with a growing body of recent research linking them to poorer clinical outcomes. In endometrial tumors, TAMs are predominantly polarized toward an M2-like phenotype, which fosters immunosuppression, supports angiogenesis, and facilitates cancer progression through secretion of anti-inflammatory cytokines and pro-angiogenic factors, as well as by promoting epithelial-mesenchymal transition and metastasis (146). Recent translational studies have shown that metabolites such as tumor-derived lactate drive M2 polarization, promoting deeper myometrial invasion and advanced disease, and that blocking key signaling axes (e.g., IL-6) can attenuate this pro-tumoral activity, highlighting novel therapeutic targets (146). Observational evidence also indicates that a high density of TAM infiltration correlates with aggressive tumor features—including higher grade, increased lymphatic invasion, and lymph node metastasis—and serves as an independent prognostic factor for reduced recurrence-free survival in patients with endometrial cancer, underscoring its clinical relevance (147). Mechanistically, M2 TAMs suppress anti-tumor immunity by inhibiting effector T-cell function and remodeling the TME toward tolerance, which can contribute to resistance against immune checkpoint blockade and other immunotherapies (148). As a result, strategies aimed at reprogramming TAMs toward a more inflammatory M1 phenotype, blocking their recruitment or key signaling pathways, or disrupting their metabolic support are under active investigation as adjuncts to improve immunotherapy responses in endometrial cancer (149).

### 6.5 Important role of the regulatory T cells

Although tumor-associated macrophages (TAMs) have been indicated here as a potential key drivers of an immunosuppressive tumor microenvironment (TME), we should also explicitly discuss regulatory T cells (Tregs), another major immunosuppressive population that profoundly impacts cancer immunity and immunotherapy outcomes. Tregs, typically defined by high expression of CD4 and the transcription factor FoxP3, accumulate within many solid tumor TMEs where they suppress effector T-cell functions through multiple mechanisms including secretion of immunosuppressive cytokines (e.g., IL-10 and TGF-β), high CD25-mediated IL-2 consumption, and direct cell-cell suppression, thereby facilitating tumor immune evasion and correlating with resistance to checkpoint blockade therapies (150) (22). Unlike innate immune cells, Tregs are adaptive CD4^+^ lymphocytes whose enriched presence within tumors is associated with dampened anti-tumor immunity and poor clinical responses in various cancer types, underscoring their relevance to immunotherapeutic strategies (151). Moreover, recent evidence suggests that TAMs and Tregs can cooperate metabolically and functionally within the TME to reinforce immunosuppression, with certain metabolites promoting both the protumoral polarization of TAMs and the suppressive activity of Tregs, creating a synergistic barrier to effective cytotoxic T-cell responses (152).

Beyond mere enumeration, mentioning Treg biology is critical because targeted modulation of Treg, for example, through selective depletion or reprogramming of their suppressive phenotype, has emerged as a promising avenue to enhance the efficacy of cancer immunotherapy, including in tumors such as endometrial carcinoma where immune contexture can influence therapeutic responsiveness (153). Therefore, integrating both TAM and Treg dynamics offers a more comprehensive view of the immunosuppressive networks that must be overcome to optimize clinical benefit (154).

### 6.6 Clinical, logistical, financial and systemic challenges of immunotherapy

Immunotherapy encounters various challenges on multiple levels, including clinical, logistical, financial, and systemic. Developing immunotherapies that are consistently effective across a majority of patients and cancer types remains a significant challenge (155). While some patients show dramatic results, many treatments are only effective in a selected group of cancers and often in a minority of patients (154, 155). Variability in patient response depends on factors such as the need for more biomarkers and identified cancer pathways, tumour heterogeneity, cancer type and stage, treatment history, and the immunosuppressive biology of the cancer (155).

Moreover, clinical efficacy of immunotherapy is hampered by the development of resistance in patients, with genetic and epigenetic alterations in tumour cells modulating immune checkpoint molecules, resulting in the escape of immune surveillance (154, 155).

Another major limitation of cancer immunotherapy is the availability of known targetable tumour-specific antigens, also called “neoantigens,” that are solely expressed by tumour cells (155). Furthermore, tumour microenvironment can orchestrate an immunosuppressive environment, weakening the immune response and promoting tumour progression (156). The same TME features that impair nanomedicine delivery can also cause immunosuppression (157).

A further difficulty arises from the complexity of cancer, tumour heterogeneity, and immune escape as well as the lack of definitive biomarkers for assessing clinical efficacy of cancer immunotherapies (154, 155). Then, there is the question of drug delivery issues related to the short half-life of agents, on-target/off-tumour toxicity, cytokine release syndrome (CRS), and neurotoxicity (158).

Also, absence of optimized clinical study designs to determine efficacy and differences between response patterns to cytotoxic agents and immunotherapies as well as limitations of current animal models to predict the efficacy of cancer immunotherapy strategies in humans remain a hurdle to tackle (154, 157).

**Figure 3:**
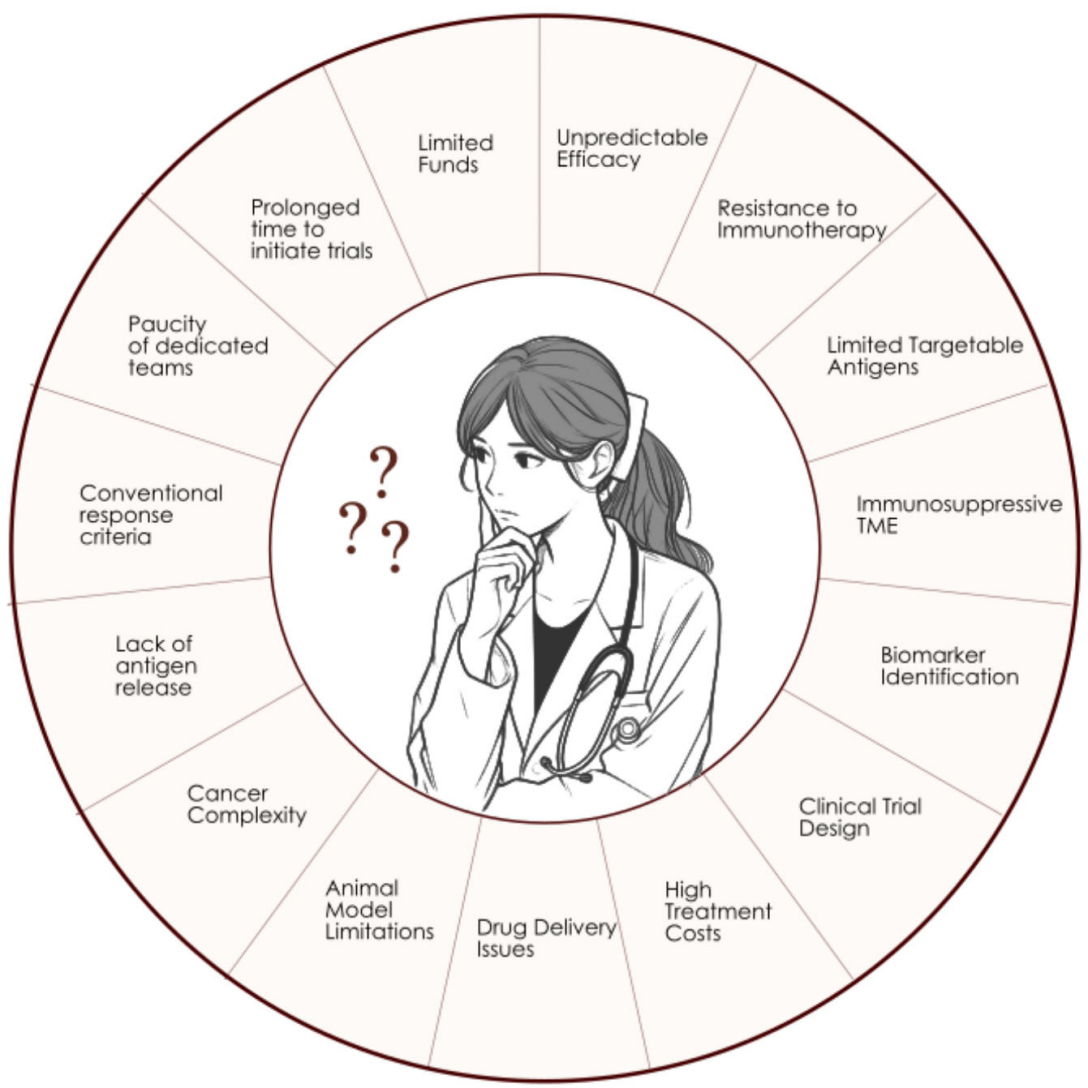
Challenges of immunotherapy.

And last but not least, high treatment costs associated with cancer immunotherapies create a financial obstacle which is not always easy to overcome (155). The key points of the discussed challenges have been summarized and presented in **Figure 3**.

## 7 Hopes for the future - new therapies and interesting clinical trials

Currently, there are over 500 clinical trials focused on endometrial cancer, with more than 400 actively recruiting participants or preparing to commence recruitment. These trials investigate diverse aspects of endometrial cancer treatment, including the efficacy of novel drug combinations, advancements in immunotherapy, and strategies for fertility preservation.

The RAINBO program is an international platform dedicated to personalizing adjuvant treatment for endometrial cancer based on molecular profiling (159). Its primary goals are to enhance cure rates through the addition of novel targeted therapies or to reduce treatment toxicity and improve quality of life through treatment de-escalation. By focusing on predictive and prognostic biomarkers, the program aims to refine both prognostication and treatment allocation. It encompasses four international clinical trials alongside an overarching research initiative.

The p53abn-RED trial is a randomized phase III study comparing adjuvant chemoradiation followed by two years of olaparib to chemoradiation alone for stage I–III p53abn endometrial cancer (159). The MMRd-GREEN trial, another phase III study, evaluates the combination of radiotherapy with concurrent and adjuvant durvalumab for one year versus radiotherapy alone in stage II (with lymphovascular space invasion - LVSI) or stage III mismatch repair-deficient endometrial cancer (160). The NSMP-ORANGE trial focuses on treatment de-escalation, comparing radiotherapy followed by two years of progestin to chemoradiation for women with oestrogen receptor-positive stage II (with LVSI) or stage III no specific molecular profile endometrial cancer (161). Finally, the POLEmut-BLUE trial, a phase II study, investigates the safety of adjuvant therapy reduction in stage I–III POLEmut endometrial cancer, ranging from no adjuvant therapy for lower-risk disease to no therapy or radiotherapy alone for higher-risk disease (161). The program’s overarching research component integrates data and tumour material from all participants to conduct translational research, assessing the efficacy, toxicity, quality of life, and cost-utility of molecular class-based adjuvant therapies (159).

Fertility-preserving treatments are an important area of research in endometrial cancer, particularly for patients with atypical endometrial hyperplasia or early-stage disease who wish to retain their ability to have children. The study “Value of Levonorgestrel-Releasing Intrauterine System (LNG-IUS) in the Fertility-Preserving Treatment of Atypical Endometrial Hyperplasia and Early Endometrial Carcinoma” investigates the effectiveness of the LNG-IUS in achieving this goal. By assessing key outcomes such as pathological response, pregnancy rates, and live birth rates, the trial aims to provide valuable insights into the potential of LNG-IUS as a fertility-sparing treatment option (162).

For cancer survivors, lifestyle and empowerment techniques play a crucial role in managing long-term health and well-being. The LETSGO trial in Norway is evaluating a new follow-up model for gynecologic cancer survivors, including those with endometrial cancer. This study compares traditional follow-up care to an alternative approach based on self-management interventions, supported by a smartphone application to help patients manage the physical and mental late effects of cancer treatment. The trial addresses the evolving needs of cancer survivors, as improvements in treatment have led to longer survival times, often with age-related comorbidities. To meet these needs, the research group has developed an evidence-based, risk-stratified follow-up model, offering one or three years of hospital follow-up depending on the patient’s risk level, with a focus on improving coping strategies and managing late effects without increasing healthcare costs (162).

## 8 Conclusions

The current treatments of endometrial cancer worldwide include surgery, radio-, chemo- and hormonal therapy, most of which have been developed decades ago. Nevertheless, emerging therapies focusing on targeted approaches like immunotherapy show great promise for personalized care and offer multiple treatment solutions. Immune checkpoint inhibitors blocking immune suppression and allowing T lymphocytes to attack tumour cells, include nivolumab, pembrolizumab, and dostarlimab (PD-1 inhibitors) as well as atezolizumab, avelumab, and durvalumab (PD-L1). Cancer vaccines’ therapeutic profile enhances the body’s adaptive immune system response to malignant cells. Naturally, they have a role not only in treating but also in preventing recurrence of endometrial cancer. There is also a growing number of studies focusing on the development of oncoviral therapies. Adaptive cellular therapy refers to the use of modified immune cells, such as chimeric antigen receptor (CAR) T-cells, to enhance the immune system’s ability to target and destroy cancer cells. The therapy is very promising but further clinical trials are necessary to evaluate.

## Glossary

ALPP: Anti-alkaline phosphatase placental
APC: Adenomatous polyposis coli
ARID1A: AT-rich interactive domain-containing protein 1A
ATR: Ataxia telangiectasia and Rad3 related
BSABS: Bispecific antibodies
CAR: Chimeric antigen receptor
CAR-T-MESO: Chimeric mesothelin antigen receptor-modified T
CD: Cluster of differentiation
CI: Confidence interval
CLDN6: Claudin 6
CRS: Cytokine release syndrome
CTNNB1: Catenin beta 1
CXCL12: Chemokine 12
DC: Dendritic cell
DFS: Disease-free survival
DMMR: Mismatch repair deficiency
EC: Endometrial cancer
EMA: European Medicines Agency
EPCAM: Epithelial cell adhesion molecule
ERA: Estrogen receptor alpha
ESMO: European Society of Medical Oncology
EU: European Union
FDA: Food and Drug Administration
FBP: Folate-binding protein
FGFR2: Fibroblast growth factor receptor 2
HGF: Hepatocyte growth factor
HER2: Human epidermal growth factor receptor 2
HPV: Human papillomavirus
HR: Hazard ratio
IGG4: Immunoglobulin G subclass 4
IGFS: Insulin-like growth factors
IL: Interleukin
KRAS: Kirsten rat sarcoma virus
LKB1: Liver kinase B1
LNG-IUS: Levonorgestrel-releasing intrauterine system
LVSI: Lymphovascular space invasion
MA: Megestrol acetate
MDSCS: Myeloid-derived suppressor cells
MMRP: Mismatch repair protein
MPA: Medroxyprogesterone acetate
MRA: Medical Research Agency
MSI: Microsatellite instability
MSI-H: High microsatellite instability
MSS: Microsatellite stability
MTOR: Mammalian target of rapamycin
MUC16: Mucin-16 (ovarian cancer-related tumor marker CA125)
NK: Natural killer
ORR: Overall response rate
PCOS: Polycystic ovary syndrome
PFS: Progression-free survival
PD-L1 (2): Programmed death-ligand 1 (2)
PI3K: Phosphoinositide 3-kinases
PIK3CA: Phosphatidylinositol-4,5-bisphosphate 3-kinase catalytic subunit alpha
PIK3R1: Phosphoinositide-3-kinase regulatory subunit 1
POLE: Polymerase (DNA directed), epsilon, catalytic subunit
PR: Progesterone receptor
SCNA: Somatic copy number alterations
SERMS: Selective estrogen receptor modulators
TAMS: Tumour-associated macrophages
TGF-β: Tumor growth factor beta
TGCA: Tumor growth factor beta
TILS: Tumour-infiltrating lymphocytes
TME: The tumour microenvironment
TREGS: Regulatory T cells
TSAS: Tumour-specific antigens
VEGF: Vascular endothelial growth factor
VV: Vaccina virus
